# Oral hygiene, quality of life, and risk of heart failure

**DOI:** 10.3389/froh.2025.1438026

**Published:** 2025-03-05

**Authors:** Jeffrey J. VanWormer, Neel Shimpi, Kelly Schroeder, Arin VanWormer, Gaurav Jain, Richard A. Dart

**Affiliations:** ^1^ Center for Clinical Epidemiology & Population Health, Marshfield Clinic Research Institute, Marshfield, WI, United States; ^2^ Center for Dental Benefits, Coding and Quality, American Dental Association, Chicago, IL, United States; ^3^ Analytics and Data Insights, CareQuest Institute for Oral Health, Boston, MA, United States; ^4^ Department of Nursing, University of Wisconsin–Eau Claire, Eau Claire, WI, United States; ^5^ Marshfield Dental Center, Family Health Center of Marshfield, Marshfield, WI, United States; ^6^ Center for Precision Medicine Research, Marshfield Clinic Research Institute, Marshfield, WI, United States

**Keywords:** oral hygiene, oral health, quality of life, heart failure, adults, USA

## Abstract

**Purpose:**

Heart failure (HF) is a debilitating form of cardiovascular disease that is increasing worldwide. Poor oral health is an established risk factor for cardiovascular disease, but there are few studies specific to the development of HF. In particular, there are no known studies on oral hygiene and HF in the United States. This study characterizes the association between oral hygiene, oral health-related quality of life (OHRQoL), and risk of HF in adults.

**Methods:**

A case-control sample was assembled from adult patients of the Marshfield Clinic Health System in north-central Wisconsin. HF cases were matched on age and sex to HF-free controls. HF case status, along with clinical covariates, were extracted from electronic health records. Surveys were used to collect oral health exposures (toothbrushing, flossing, dental visits, and OHRQoL) and other sociodemographic covariates. Multivariable regression was used to examine associations with HF.

**Results:**

Survey response rates were 67% in HF cases and 74% in HF-free controls, yielding an analytical sample of 410 individuals. OHRQoL was not significantly associated with HF, but both oral hygiene and last dental visit were. Specifically, multivariable models revealed that participants with excellent oral hygiene had significantly lower odds of HF as compared to those with fair/poor oral hygiene [aOR = 0.47 (CI: 0.24, 0.95), *p* = 0.035]. Similarly, participants with a more recent dental visit that occurred less than two years prior had significantly lower odds of HF as compared to participants with a dental visit that occurred more than two years prior [aOR = 0.43 (CI: 0.25, 0.74), *p* = 0.002].

**Conclusion:**

Good oral hygiene (i.e., regular toothbrushing/flossing) and a recent dental visit were protective against HF. If poor oral health is established as a causal contributor to HF in future research, it could open up previously unrecognized or underappreciated additional pathways to prevention whereby the risk of HF development could be interrupted by more intense screening/recognition of deteriorating oral health by medical care teams, as well as a more direct focus on cardiovascular disease prevention by dental care teams.

## Introduction

There are nearly seven million U.S. adults living with heart failure (HF) today (1) and this number is projected to increase nearly 50% by 2030 (2). HF often follows ischemic vascular disease and/or hypertension (3), and the increase in HF prevalence in recent decades is primarily attributed to an aging population and improved cardiovascular disease (CVD) treatment and survival (4). HF underlies one of every eight deaths annually in the U.S (5)., and about half of all HF patients will die within five years of their initial diagnosis (6, 7).

In contrast to HF, oral diseases are less common in the U.S. today compared to decades ago (8), though they remain common at older ages. Poor oral health is a well-established independent risk factor for multiple health conditions, including several forms of CVD (9), but there is limited research on links between oral health and HF specifically. The biological basis for such a link is centered on diminished endothelial function in blood vessels due to systemic inflammation (10, 11), with the maintenance of said inflammation partially driven by the host's immune response to persistent bacteremia from the oral cavity (12). Direct evidence of this link is limited, but severe periodontitis was found to be more common in a convenience sample of German adults with HF as compared to a historical sample of the general population (13). Tooth loss is perhaps the clearest oral health-related correlate of HF. A large prospective cohort study in Australia found that adults who reported having no teeth had a nearly two-fold increase in the odds of incident HF over four years relative to those with ≥20 teeth (14). In Swedish adults with periodontitis, each retained tooth was associated with a 13% lower rate of HF incidence over 16 years (15). Similarly, in a large cohort of South Korean adults, each missing tooth was associated with a nearly 2% increase in the rate of incident HF over 8 years (16).

Despite the growing acknowledgement that poor oral hygiene impacts the development of CVD, the evidence base regarding HF remains limited. Some previous studies in this field had large samples, but many relied on self -reported or administratively indicated HF status. Others did not account for potential confounders such as smoking or diabetes. While tooth loss (14–16) and periodontitis (13, 17, 18) links have received prior research attention, there are no known studies of oral hygiene links to HF in research samples from the U.S. The objective of this study was to examine the association between oral hygiene, oral health related quality of life (OHRQoL), and the risk of HF in adults residing in Wisconsin.

## Methods

### Design and setting

This study utilized a case-control design that combined survey and electronic health records (EHR) data. The source population from which HF cases and HF-free controls, as defined below, were identified and recruited from included Marshfield Clinic Health System [MCHS; headquarters Marshfield, WI (USA)] patients in the Marshfield Epidemiologic Study Area (MESA). MCHS is an integrated healthcare system that serves small and midsize communities across northern and central Wisconsin, and the upper peninsula of Michigan. MESA is a subset of the MCHS patient population, which serves as a research resource that tracks person-time observation windows of patients who reside in ZIP codes within MCHS primary service areas (19, 20).

### HF cases and HF-free controls

Two groups were compared; HF cases vs. a group of age- and sex-matched HF-free controls. Specifically, HF cases included living individuals in the source population who were: (1) age 35–84 years, and (2) per a validated electronic phenotyping algorithm that considers structured and unstructured EHR data (21), presented for a first (confirmed) HF diagnosis at a MCHS facility within the previous year. Patients were excluded if they did not have an MCHS care visit within the prior year, could not read or respond to the English language survey, resided at a known institution (e.g., medical, penal), or were under age 35 or over age 84 were excluded to minimize the complexities of HF causal factors in very young and very old age groups, who may be more likely to have congenital heart defects or severe multi-morbidity. Prior to study invitation, HF case status was confirmed by a manual review of medical chart information to verify consistency with the Framingham HF criteria (22), and to ensure cases had indeed been newly diagnosed with HF. For each enrolled HF case, we aimed to also enroll two randomly selected HF-free controls, frequency matched by sex and age groups (35–64, 65–74, and 75–84 years). As a precise sample size calculation was not possible due to the lack of prior data on oral hygiene and HF associations in the U.S., all known study-eligible HF cases were invited during the 6-month recruitment period (November 2022 through April 2023). The MCHS Institutional Review Board approved all study procedures in advance, including a request to waive documentation of informed consent and HIPAA authorization for survey participants.

### Recruitment procedures

Recruitment was conducted in regular waves over a 6-month timeframe. Study-eligible HF cases were randomly selected for invitation each week. Once a given HF case completed the study survey, a random sample of up to four matched HF-free controls was selected for invitation. Contact information for enumerated individuals was extracted from MCHS administrative records. Each enumerated individual received the following outreach efforts: (1) mailed invitation packet, which included a cover letter, study information sheet, survey instrument (with option to complete electronically), return mailer, and $5 cash incentive; (2) mailed reminder/thanks postcard; (3) follow-up telephone calls (up to three attempts) for non-respondents (plus a verbal survey response option); and (4) final mailed follow-up packet, which included the same elements as the invitation packet. By completing the survey, participants consented to have their survey data linked to their EHR data for study analyses.

### Oral health and covariates

The primary exposures were markers of oral health, as measured by a self-report survey using three separate variables, including oral hygiene, last dental visit, and OHRQoL. An oral hygiene status indicator was used that considered both toothbrushing and flossing frequency (i.e., How often do you brush your teeth?, How often do you floss your teeth?), as has been used elsewhere (23). Specifically, the oral hygiene status indicator included three ordinal categories; excellent, good, and fair/poor. This exposure is described in more detail by VanWormer and colleagues (23), but briefly, respondents with “excellent” oral hygiene report brushing their teeth ≥2 times per day and flossing daily. Those with “good” oral hygiene report brushing their teeth once daily and flossing daily or most days, or brushing their teeth ≥2 times per day and flossing some or most days. All others are categorized as having “fair/poor” oral hygiene. These general recommendations were based on the American Dental Association guidelines of optimal frequencies of toothbrushing and flossing (24) and are supported by other studies that observed links between oral hygiene and cardiovascular disease risk factors (23, 25). The last dental visit was dichotomized as having occurred within two years or having occurred longer than two years prior (i.e., About how long has it been since you last saw a dentist?). To assess OHRQoL, participants also completed the brief Oral Health Impact Profile (OHIP-5) (26, 27). The OHIP-5 is a 5-item instrument that asks participants to rate their experiences with four domains of common oral problems found in the longer version of the OHIP surveys, including oral function, orofacial pain, orofacial appearance, and psychosocial impact. Results from the OHIP-5 are scored on a 0–20 point scale, with the higher scores indicating lower OHRQoL. Smoking status (i.e., Which of the following best describes when you most recently smoked cigarettes?) and education levels (i.e., What is the highest level of education that you have completed?) were collected from the study survey. Other covariates, including age, gender, race/ethnicity, Medicaid status, number of ambulatory care visits (in past three years), body mass index (BMI), and prevalent type 2 diabetes (28), were collected from the EHR.

### Analyses

Analytical procedures were conducted using SAS Version 9.4 (Cary, NC). Sociodemographic characteristics were compared between HF cases and HF-free controls, and logistic regression was used to examine associations between oral health exposures and HF case-control status. Specifically, univariate models were first created to gauge the crude relationship between each oral health exposure and HF, separately. Multivariable models using PROC LOGISTIC were then fit by conditioning on the matched variables, age and sex, as well as adjusting for other covariates, including education, Medicaid status, smoking status, BMI, and type 2 diabetes.

## Results

There were 507 study-eligible individuals invited to complete the survey. Among HF cases, 135 (67%) of 203 invitees responded. Among HF-free controls, 275 (74%) of 374 invitees responded. Respondents and non-respondents were similar on known characteristics from the EHR, except for Medicaid status. Significantly fewer respondents were on Medicaid (11%) as compared to non-respondents (23%) (*p* < 0.001). As outlined in Table 1, the case and control groups were generally similar. Overall, participants had a mean age of 69 years, 60% were male, and 97% were White, non-Hispanic. Relative to HF-free controls, a significantly greater proportion of HF cases had type 2 diabetes, were current or former smokers, or had a high school (or less) level of education.

**Table 1:** Characteristics of heart failure (HF) cases and HF-free controls in Wisconsin.

Characteristics	HF cases *n* = 135	HF-free controls *n* = 275	*p*
Age (years)	69.3 ± 10.3	68.2 ± 10.5	0.333
Gender			
Female	56 (41%)	109 (40%)	0.720
Male	79 (59%)	166 (60%)	
Race/ethnicity			
White, non-Hispanic	130 (96%)	268 (97%)	0.513
Non-White or Hispanic	5 (4%)	7 (3%)	
Education			
Bachelors degree or higher	18 (13%)	54 (20%)	0.005
Associates degree or some college	38 (28%)	103 (37%)	
High school or less	68 (50%)	111 (40%)	
Unknown	11 (8%)	7 (3%)	
Medicaid	18 (13%)	26 (9%)	0.233
Ambulatory care visits (prior 3 years)	21.0 ± 13.2	11.1 ± 8.5	<0.001
Body mass index (percentile categories)			
Normal weight	17 (13%)	48 (17%)	0.337
Overweight	46 (34%)	98 (36%)	
Obese	72 (53%)	129 (47%)	
Smoking status			
Current smoker	22 (16%)	33 (12%)	0.042
Former smoker	55 (41%)	89 (32%)	
Never smoker	58 (43%)	153 (56%)	
Type 2 diabetes	50 (37%)	70 (25%)	0.015
Oral hygiene			
Excellent	15 (21%)	57 (79%)	0.005
Good	47 (30%)	112 (70%)	
Fair/poor	73 (41%)	106 (59%)	
Last dental visit			
<2 years ago	46 (34%)	43 (16%)	<0.001
≥2 years ago	89 (66%)	232 (84%)	
Oral Health Impact Profile – 5 (0–20 points)	2.1 ± 3.0	2.1 ± 2.9	0.988

Values are reported as mean ± SD or frequency (% of total).

**Figure 1:**
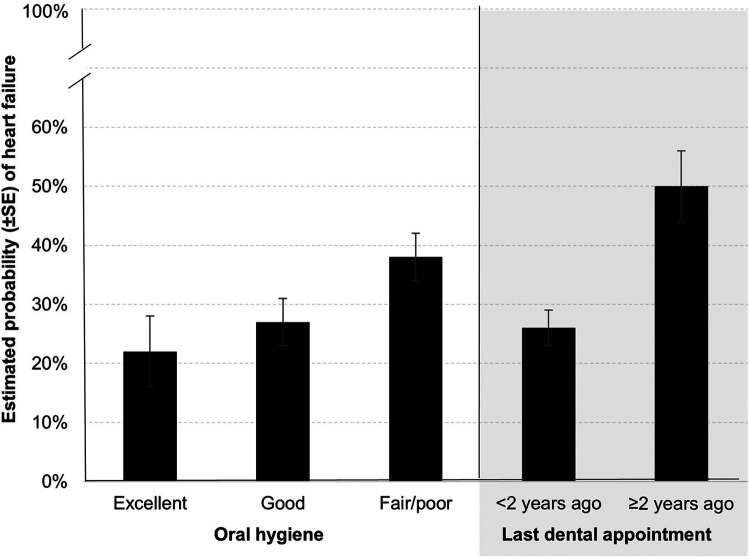
Model-estimated probability of heart failure by oral hygiene status and last dental appointment in a case-control sample of Wisconsin adults age 35–84 years.

The OHIP-5 score was not associated with HF in a univariate model (odds ratio [OR] = 1.01 [95% confidence interval (CI): 0.93–1.07], *p* = 0.988), thus no further multivariable modeling was done on that exposure. Both oral hygiene status (*p* = 0.006) and last dental visit (*p* < 0.001) were significantly associated with HF in univariate models (not shown). The detailed multivariable models of these associations are outlined in Table 2. After adjustment for age, sex, education, Medicaid, number of ambulatory care visits, smoking, BMI, and type 2 diabetes, participants with excellent oral hygiene [aOR = 0.45 (CI: 0.21, 0.95), *p* = 0.037] had significantly lower odds of HF as compared to those with fair/poor oral hygiene. Similarly, participants with a more recent dental visit that occurred less than two years prior [aOR = 0.35 (CI: 0.19, 0.63), *p* = 0.001] had significantly lower odds of HF as compared to participants with a dental visit that occurred more than two years prior. To better illustrate the multivariable associations, the model-predicted probability of HF in the analytical sample, by both oral hygiene level and last dental visit, is outlined in Figure 1.

**Table 2:** Multivariable logistic regression model of the association between heart failure (HF), oral hygiene (model 1), and last dental visit (model 2), along with covariates, in Wisconsin adults (*N* = 410).

Exposures	Model 1: HF	Model 2: HF
Oral hygiene		
Excellent	0.45 (0.21, 0.95), *p* = 0.037	—
Good	0.61 (0.36, 1.05), *p* = 0.073	
Fair/poor	— ref. —	
Last dental visit		
<2 years ago	—	0.35 (0.19, 0.63), *p* = 0.001
≥2 years ago		— ref. —
Age (years)	0.99 (0.97, 1.02), *p* = 0.605	0.99 (0.97, 1.02), *p* = 0.508
Sex		
Female	1.11 (0.66, 1.84), *p* = 0.698	1.06 (0.64, 1.76), *p* = 0.815
Male	— ref. —	— ref. —
Education		
Bachelors degree or higher	0.76 (0.37, 1.54), *p* = 0.439	0.74 (0.37, 1.49), *p* = 0.399
Associates degree or some college	0.71 (0.41, 1.22), *p* = 0.215	0.72 (0.42, 1.25), *p* = 0.247
Unknown	2.34 (0.75, 7.29), *p* = 0.142	2.01 (0.58, 7.04), *p* = 0.274
High school or less	— ref. —	— ref. —
Medicaid		
Yes	1.24 (0.55, 2.78), *p* = 0.610	1.17 (0.51, 2.66), *p* = 0.713
No	— ref. —	— ref. —
Ambulatory care visits (prior 3 years)	1.10 (1.07, 1.13), *p* < 0.001	1.10 (1.07, 1.13), *p* < 0.001
Smoking status		
Current smoker	1.47 (0.71, 3.06), *p* = 0.305	1.23 (0.58, 2.63), *p* = 0.592
Former smoker	1.36 (0.80, 2.33), *p* = 0.262	1.27 (0.74, 2.20), *p* = 0.386
Never smoker	— ref. —	— ref. —
Body mass index (percentile categories)		
Normal weight	1.00 (0.49, 2.05), *p* = 0.998	0.94 (0.46, 1.92), *p* = 0.858
Overweight	0.79 (0.46, 1.37), *p* = 0.403	0.77 (0.44, 1.34), *p* = 0.357
Obese	— ref. —	— ref. —
Type 2 diabetes		
Yes	1.09 (0.64, 1.85), *p* = 0.765	1.02 (0.59, 1.75), *p* = 0.948
No	— ref. —	— ref. —

Values are reported as odds ratio (95% confidence interval) and *p*-value of HF, relative to the reference category for categorical exposures or a 1-unit increase for continuous exposures.

## Discussion

Study findings indicated that two markers of oral health, good oral hygiene (i.e., regular toothbrushing and flossing) and a recent dental visit within the last two years, were protective against HF development. These associations were robust, even after adjustment for potentially confounding covariates such as smoking and diabetes (whose influence on HF associations were tempered in multivariable models). Our findings are consistent with several other recent studies outside of the U.S., including a large retrospective cohort study in South Korea that found frequent tooth brushing was associated a 12% lower risk of incident HF, and that a professional dental cleaning in the past year was associated with a 7% reduction of HF risk (29) [with similar associations also observed in patients with type 2 diabetes (30)]. In a smaller prospective cohort study in Japan, low frequency/duration toothbrushing habits were associated with a 3-fold increase in the hazard ratio for major adverse cardiovascular events, which included HF hospitalization (31).

Reducing the burden of HF is a clear public health priority (2, 6). If poor oral health is established as a causal contributor to HF, it could identify additional, previously unrecognized or underappreciated pathways to prevention whereby the development of HF could be interrupted or at least delayed. In particular, the oral health risk factors in our study that were most strongly associated with HF, irregular toothbrushing/flossing and infrequent dental visits, are modifiable and generally reflect “upstream”, prevention- and behavioral-oriented elements of oral self-care. For example, medical care teams can intensify efforts to screen for and recognize deteriorating oral health in their patients. In addition, dental care teams can help their patients more clearly understand the important connections between oral care and prevention of CVD and other systemic health conditions. Finally, oral healthcare insurance coverage options could be expanded for low income individuals, alongside reminder/recall notices for annual dentist visits. Such practice- and systems-based improvements in medical and dental care quality should be examined in future intervention trials.

As stated previously, OHRQoL as measured by the OHIP-5, which is a composite assessment of current dental problems, was not associated with HF. This was somewhat surprising given the protective associations observed for oral hygiene and dental visits, but may reflect some differences in “cumulative dose” between these different oral health risk factors. For example, regular toothbrushing and flossing tend to be rather stable behaviors that reflect many years, perhaps a lifetime, of good oral hygiene habits (32). In contrast, the OHIP-5 is an inventory of current and specific oral symptoms such as mouth pain or difficulty chewing. Though OHRQoL has long been known to be enhanced/supported by good oral hygiene and regular preventive dental care (33), it does not necessarily reflect a lengthy history of poor oral health. OHIP-5 scores were generally low in our sample, indicating relatively few oral health complications present at the time of survey completion, and a more limited influence on HF. It is unclear if or how an extensive history of oral health complications would have a greater impact on the development of HF, but this could again be a subject of future investigation.

### Strengths and limitations

Strengths of this study included the objective, validated ascertainment of HF case status, matching and statistical adjustment for potential confounders, and the sampling of participants from a defined source population with complete capture of their medical care. The biggest limitation was the observational design, which precluded causal conclusions. HF cases were recently diagnosed, but were technically not incident cases since the oral health exposures were collected after (but temporally near to) the time of clinical recognition of HF. Although cases and controls were matched on basic characteristics, confounding by unmeasured exposures (e.g., tooth counts, nutrition, blood pressure control) remains possible. In addition, the oral health exposures in our study were self-reported and thus subject to recall and/or self presentation biases. These oral health exposures also lacked some important details, such as the reason for the last dental visit (e.g., routine cleaning, emergent procedure), which could be influential on HF status and thus important to cover more comprehensively in future studies. Finally, the predominantly rural and racially homogenous source population impedes broader generalizability. Future research should confirm these associations in larger, more diverse samples, alongside more objective assessments of oral health and detailed information on oral care procedures.

## Conclusions

HF is a debilitating disease that typically indicates limited remaining life expectancy (7). Although clinical care for HF is improving (34), early interventions are yet needed to help patients avoid or delay HF. Oral self-care and regular dental visits are already well established as critical elements of maintaining optimal oral health (24), but may also help prevent HF. Such components of good oral health can be more routinely screened for, encouraged, and reinforced by both medical and dental care team interventions in patients at high risk.

## Data Availability

The datasets presented in this article are not readily available because the datasets for this study are unavailable for public access because informed consent to share said data (beyond the research team) was not obtained from study participants, but de-identified data may be available from the corresponding author on reasonable request. Requests to access the datasets should be directed to vanwormer.jeffrey@marshfieldresearch.org.
